# Locating Order-Disorder Phase Transition in a Cardiac System

**DOI:** 10.1038/s41598-018-20109-6

**Published:** 2018-01-31

**Authors:** Hiroshi Ashikaga, Ameneh Asgari-Targhi

**Affiliations:** 1 Cardiac Arrhythmia Service, Johns Hopkins University School of Medicine, 600 N Wolfe Street, Carnegie 568, Baltimore, Maryland 21287 USA; 2 Division of Sleep and Circadian Disorders, Brigham and Women’s Hospital, 221 Longwood Avenue, Suite 438, Boston, Massachusetts 02115 USA

## Abstract

To prevent sudden cardiac death, predicting where in the cardiac system an order-disorder phase transition into ventricular fibrillation begins is as important as when it begins. We present a computationally efficient, information-theoretic approach to predicting the locations of the wavebreaks. Such wavebreaks initiate fibrillation in a cardiac system where the order-disorder behavior is controlled by a single driving component, mimicking electrical misfiring from the pulmonary veins or from the Purkinje fibers. Communication analysis between the driving component and each component of the system reveals that channel capacity, mutual information and transfer entropy can locate the wavebreaks. This approach is applicable to interventional therapies to prevent sudden death, and to a wide range of systems to mitigate or prevent imminent phase transitions.

## Introduction

Phase transitions between ordered and disordered states are pervasive in complex systems that consist of large numbers of interacting components^1,2^. Examples of phase transitions include stock market crashes^3^, climate changes^4^, ecological collapses^5^, population dynamics^6,7^, epileptic seizures^8^, and flocking of birds^9^. In biological systems, phase transitions are particularly common because they tend to self-organize towards criticality – the border between order and disorder^10–12^. Work on phase transitions has focused primarily on discovering early warning signs to predict when the phase transitions occur^13^. However, little attention has been paid to where in the networked dynamical system the order-disorder phase transition begins, which is at least as important as when it occurs. An imminent phase transition may be mitigated or prevented by identifying and modifying the specific components of the system responsible for the initiation of the phase transition.

In the heart, fibrillation is a disordered state of cardiac excitation that is one of the most common causes of sudden cardiac death, accounting for an estimated 15–20% of all deaths worldwide^14^. Fibrillation is frequently initiated by a train of rapid stimuli from a region of the heart other than the sinoatrial node. Common clinical examples include electrical misfiring from the pulmonary vein inducing paroxysmal atrial fibrillation^15^, or ectopic beats from the Purkinje fibers causing idiopathic ventricular fibrillation^16^ in structurally normal hearts without myocardial fibrosis. Identifying the components that initiate an order-disorder phase transition from a regular heart rhythm to fibrillation enables an interventional strategy to target those culminating components to prevent morbidity and mortality resulting from atrial^17^ and ventricular fibrillation^18–20^.

Information theory can be used to quantify the structural information content of networks^21^ and locate the network component that determines the behavior of the system^22^. In addition, information-theoretic metrics such as mutual information^23^ and transfer entropy^24^ can be used to predict phase transitions in many complex systems^25–31^; however, the applicability of these metrics in complex systems has two significant limitations. First, these metrics require measuring the time series history of all the components in the system, which is virtually intractable in real-world systems. Second, due to the large number of components, it is not trivial to calculate those information-theoretic measures between all-to-all pairs of components in the system.

The aim of the present study was to develop a computationally efficient, information-theoretic approach to predict the locations of an order-disorder phase transition from a regular heart rhythm to fibrillation in a cardiac system with no structural abnormality. Because fibrillation is initiated by a wavebreak – an intersection between the wavefront (action potential upstroke) and the waveback (action potential repolarization) of electrical traveling waves^32^ – we predicted the locations of phase transition based on our information-theoretic approach, and compared those locations with the measured locations of wavebreaks.

To simulate the common clinical examples of initiation of fibrillation, we considered a cardiac system where the system behavior is controlled by one single driving component that fires a train of rapid stimuli. In this setting, mutual information quantifies shared information, or the joint probability distribution (macrostate) over the possible microstates, between the driving component and each component of the system. Transfer entropy quantifies information flow from the driving component to each component of the system. In essence, our approach obviates the need for accounting for interactions between all-to-all pairs of components in the system, but instead only requires a communication analysis between the driving component and each component of the system, which is computationally feasible in real-world cases^33^.

Fibrillation is often preceded by alternating action potential duration (APD) called APD alternans^34,35^, an intrinsic oscillatory dynamics of cardiac cells that arises out of a period-doubling bifurcation in the stochastic dynamics of intracellular calcium cycling^36–38^. The emergence of APD alternans could be considered as an order-disorder phase transition^39^, and earlier small clinical studies showed that it may be associated with sudden cardiac death^40^. However, a recent larger multicenter clinical trial concluded that APD alternans does not predict sudden cardiac death^41^. In the present study, although we analyzed APD alternans as a key precursor to a phase transition, we focus on the emergence of fibrillation as an order-disorder phase transition because it is more clinically relevant^42^.

## Results

### Conceptual overview

**Figure 1:**
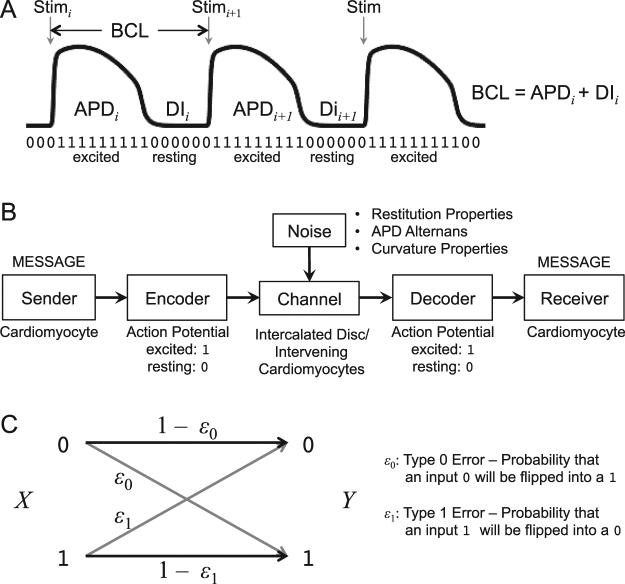
Information-theoretic analysis of cardiac dynamics. (**A**) *Cardiac action potential*. Three cardiac action potentials in response to regular stimuli with an external current (‘*Stim*’) are shown. The basic cycle length (*BCL*) is the interval between regular stimuli. The action potential duration (*APD*) is measured at 90% repolarization (*APD*_90_). The *i*th diastolic interval (*DI*_*i*_) is defined as the difference between BCL and the *i*th APD (*APD*_*i*_). The microstate of each cardiac component is encoded as 1 when excited (during APD) or 0 when resting (during DI). (**B**) *Heart as a communication system*. The cardiomyocytes act as an information source/encoder and a receiver/decoder with a channel being intercalated discs/intervening cardiomyocytes. Intrinsic dynamic cardiac properties such as restitution properties, APD alternans, and curvature properties serve as noise in the channel that could potentially introduce communication error. Figure modified from^23^. (**C**) *Binary asymmetric channel*. The channel has a probability *ε*_0_ that an input 0 will be flipped into a 1 (type 0 error) and a probability *ε*_1_ for a flip from 1 to 0 (type 1 error).

We described the microstate of each cardiac component as either 1 (excited) or 0 (resting) in a binary time series (Fig. 1A). We used information theory to quantify the macrostate of each component and communication between components. Intrinsic cardiac properties such as restitution properties, APD alternans, and curvature properties serve as noise in the communication channel that could potentially introduce errors (Fig. 1B). We considered these channels to be a binary asymmetric channel, the most general form of binary discrete memoryless channel, to quantify the errors and the channel capacity (Fig. 1C). Restitution properties and one-dimensional (1-D) information dynamics of the cardiac system is described in the Supporting Information (SI) Appendix 1 and 2.

### Order-disorder phase transition

**Figure 2:**
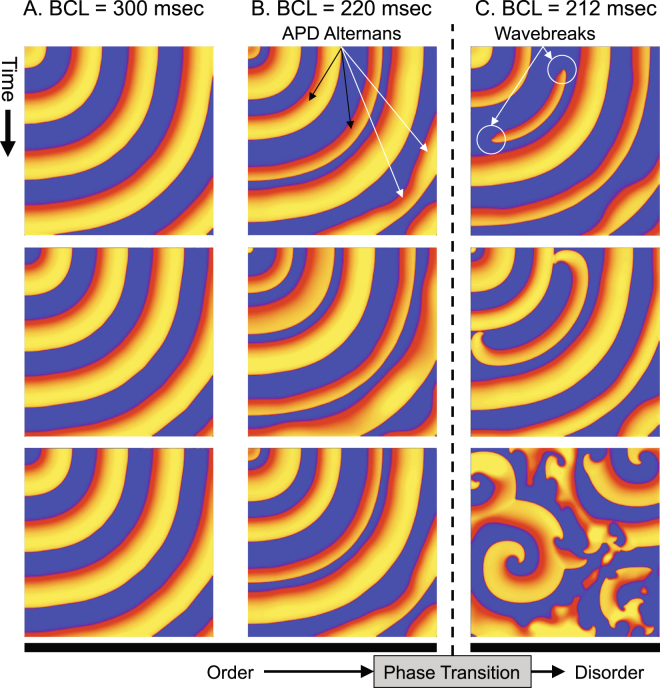
Order-disorder phase transition. The size of the lattice is 25 cm × 25 cm (Δx = 0.025 cm). The stimulus site is the top left component of the lattice, and the wave of cardiac excitation travels radially over the lattice. (**A**) *BCL* = *300* *msec* (*normal APD response*). (**B**) *BCL* = *220* *msec* (*APD alternans*). (**C**) *BCL* = *212* *msec* (*wavebreaks*).

We applied regular stimuli at the top left component of a two-dimensional (2-D) lattice, and used BCL as a control parameter to study the order-disorder phase transition in the cardiac system. We designed the model such that it is simple yet incorporates all of the intrinsic properties of 2-D cardiac dynamics; restitution properties, APD alternans and wavefront curvature properties (Fig. 1B)^43^. At BCL = 300 msec, each stimulus generated one action potential, and APD quickly became uniform spatially and temporally after a few stimuli (Fig. 2A, SI Movie 5). At BCL = 220 msec, APD alternans was observed because BCL was shorter than the period-doubling bifurcation (Fig. 2B, SI Movie 6). Successive traveling waves alternated with long and short APD. APD alternans was also observed within the same wave as well as between waves, because the conduction velocity of the traveling wave is a function of the local wavefront curvature – the conduction velocity becomes slower as the local curvature becomes more convex because of a source-sink mismatch^44^. This property made the conduction velocity at the top and the left border of the lattice higher than that of the diagonal direction. In addition, this property made the conduction velocity farther away from the stimulus site higher as the local curvature became less convex. Therefore, although the lattice is anatomically isotropic, it is functionally anisotropic depending on the wavefront curvature. At BCL = 212 msec, partial conduction block occured at the top and the left edges of the wave, creating wavebreaks (Fig. 2C, SI Movie 7). This is because the conduction velocity of these portions of the wave was higher than the rest of the wave. The wavebreaks initiated spiral waves that became completely out of sync with the stimuli at the top left corner of the lattice. Once initiated, the spiral waves broke up into more spiral waves that persisted until the end of the observation period. Such disordered states were observed at BCL equal to and shorter than 218 msec. In this study we defined this sudden change in system behavior as an order-disorder phase transition.

### Information dynamics

**Figure 3:**
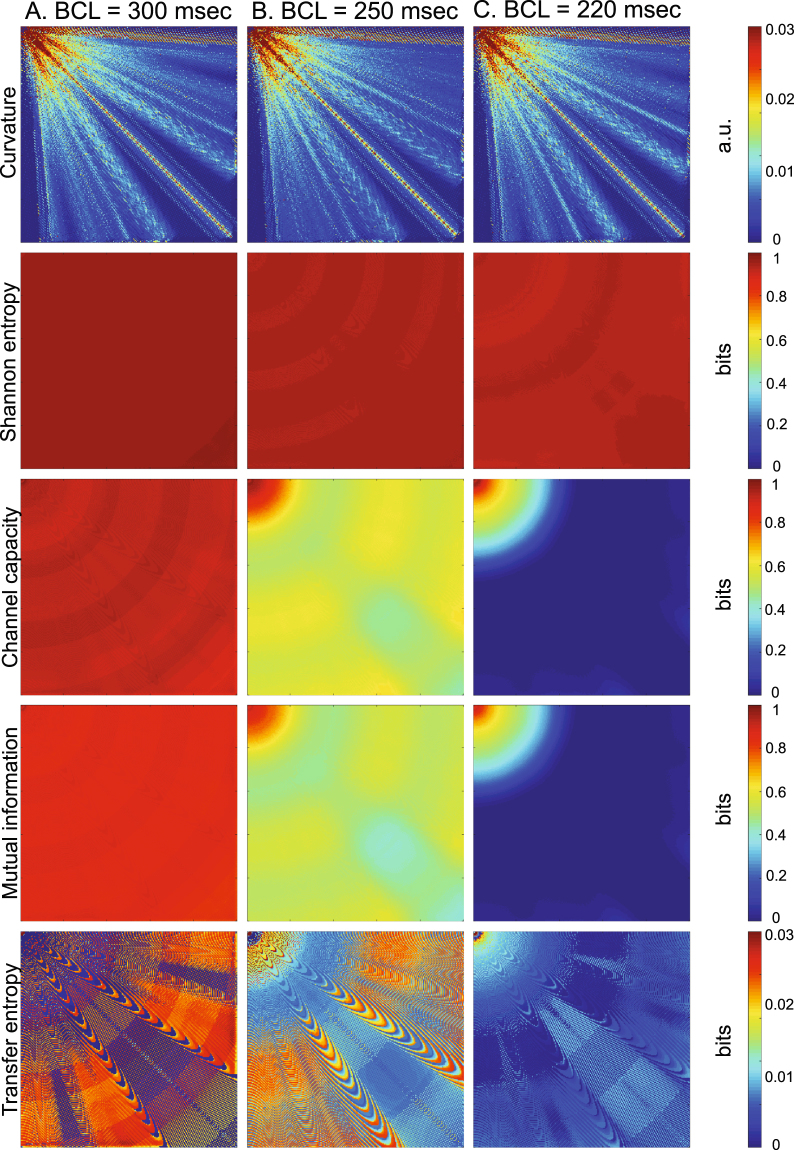
Information dynamics. Columns: (**A**) *BCL* = *300* *msec* (*normal response*). (**B**) *BCL* = *250* *msec* (*mild APD alternans*). (**C**) *BCL* = *220* *msec* (*moderate APD alternans*). Rows: (from top to bottom) curvature (a.u.; arbitrary units), Shannon entropy (bits), channel capacity (bits), mutual information (bits), and transfer entropy (bits).

Wavefront curvature was regionally heterogeneous in the 2-D lattice (Fig. 3, ‘curvature’). Curvature was highest in the vicinity of the stimulus site, and declined farther away from the stimulus site as it became less convex. In addition, curvature was lowest at the top and left border of the lattice because of the boundary effect. Importantly, curvature remained relatively invariant across different BCL. Shannon entropy also remained relatively invariant across different BCL, confirming little change in cardiac macrostate at different BCL (Fig. 3, ‘Shannon entropy’). As BCL decreased, channel capacity significantly declined because of discordant (*out*-*of*-*phase*) alternans^45^ except in the immediate vicinity of the stimulus site (Fig. 3, ‘Channel capacity’). Concordant (*in*-*phase*) alternans^45^ was not observed because the lattice was too small for it to appear. At BCL = 220 msec, channel capacity of the vast majority of the lattice was zero. Mutual information followed the trend of the channel capacity, with only slightly lower values (Fig. 3, ‘Mutual information’). Transfer entropy was more heterogeneous even at BCL = 300 msec, and showed more complex dynamics than other information metrics (Fig. 3, ‘Transfer entropy’). In some regions, transfer entropy was low (dark blue color) at BCL = 300 msec, which went up at BCL = 250 msec (light blue color), then went down again at BCL = 220 msec. This suggests that there is a complex and dynamic information transaction between the stimulus site and individual cardiac components.

### Spatial heterogeneity of communication

**Figure 4:**
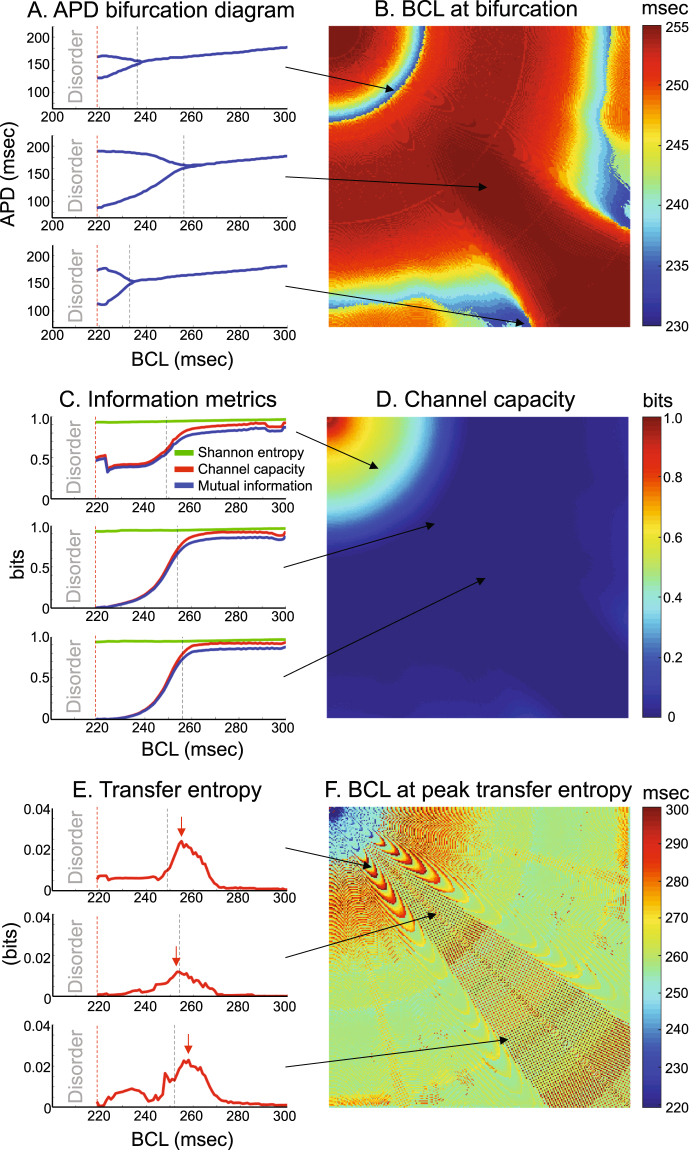
Spatial heterogeneity of communication. (**A**) *APD bifurcation diagram*. Dashed black lines indicate a period-doubling bifurcation at each component (BCL = 230–255 msec). Dashed red lines indicate an order-disorder phase transition (BCL = 218 msec). (**B**) *BCL* (*msec*) *at which period*-*doubling bifurcation occurs*. Note BCL at bifurcation is spatially heterogeneous although the system is anatomically homogeneous. Black arrows indicate the locations of components with the bifurcation diagrams on the left panels in (**A**). (**C**) *Information metrics between the stimulus site and each component*. Shannon entropy (green solid line, bits), channel capacity (red line, bits), and mutual information (blue line, bits). (**D**) *Channel capacity* (*bits*) *immediately prior to phase transition*. BCL = 219 msec. Note the channel capacity is zero (=blue) in the vast majority of components in the 2-D lattice, except in the immediate vicinity of the stimulus site. Black arrows indicate the locations of components with the diagrams on the left panels in (**C**). (**E**) *Transfer entropy from the stimulus site to each component*. Red arrows indicate the peak of transfer entropy. (**F**) *BCL at peak transfer entropy*. Note the wide range of BCL that gives the peak transfer entropy. Black arrows indicate the locations of components with the diagrams on the left panels in (**E**).

The BCL at which the period-doubling bifurcation occured was spatially heterogeneous (Fig. 4A,B). This result confirms that the system is functionally heterogeneous although anatomically homogeneous. The regions in transition between concordant APD alternans and discordant APD alternans showed shorter BCLs at bifurcation because APD tends to be stable in these regions (“*node behavior*”)^46^. Importantly, all the components in the entire lattice reached BCL at bifurcation (BCL = 230–255 msec) prior to the phase transition (BCL = 218 msec). Shannon entropy remained relatively constant across the entire range of BCL at all the components in the 2-D lattice (Fig. 4C). The period-doubling bifurcation did not have any impact on Shannon entropy. Channel capacity remained relatively flat across the higher end of the BCL range, but it began to decline as BCL declined and approached the period-doubling bifurcation.

Beyond the bifurcation, the channel capacity progressively declined and reached a plateau. The plateau was zero in the vast majority of components in the 2-D lattice, except in the immediate vicinity of the stimulus site (Fig. 4D). The behavior of the mutual information was similar to that of the channel capacity, except that the channel capacity was only slightly higher than the mutual information. Transfer entropy peaked at a wide range of BCL. Some components showed that transfer entropy peaked at BCL longer than the period-doubling bifurcation (Fig. 4E, top and bottom panels). In other components, transfer entropy peaked at BCL shorter than the period-doubling bifurcation (Fig. 4E, middle panel). Overall, BCL at which transfer entropy peaked was spatially heterogeneous in the 2-D lattice (Fig. 4F).

### Locating order-disorder phase transition

**Figure 5:**
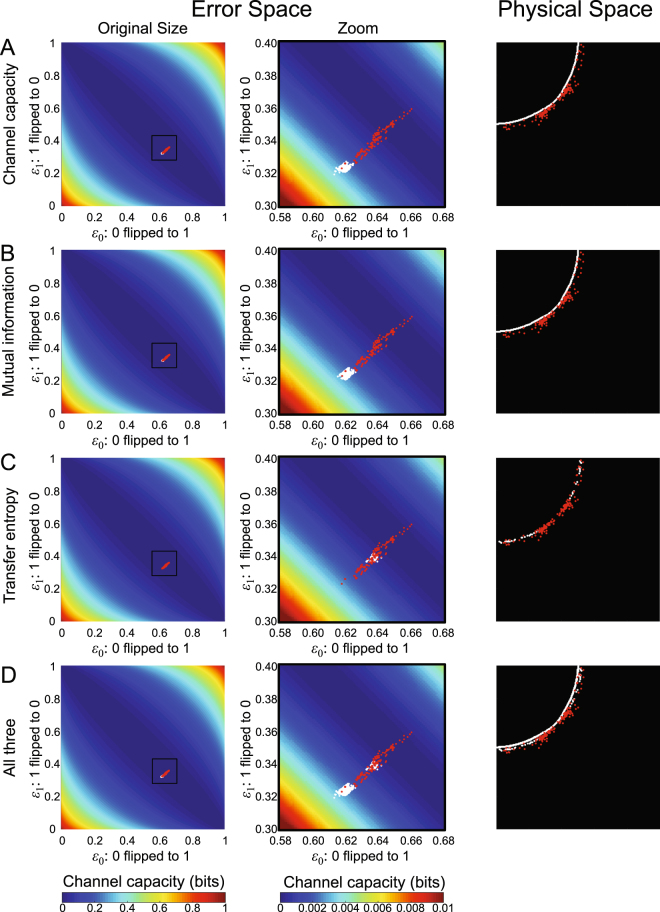
Wavebreak localization. The figure shows predicted regions of an order-disorder phase transition (white) based on information-theoretic metrics immediately prior to the phase transition (BCL = 219 msec) and actual wavebreak locations (red). The left (original size) and the middle columns (zoom) indicate the error space where the *x*-axis is the type 0 error (probability that an input 0 will be flipped into a 1), the *y*-axis is the type 1 error (probability that an input 1 will be flipped into a 0), and the color indicates the channel capacity at each location in the error space. The right column indicates the physical space of the 2-D lattice. (**A**) *Prediction based on channel capacity*. (**A**) *Prediction based on mutual information*. (**C**) *Prediction based on transfer entropy*. (**D**) *Prediction based on channel capacity*, *mutual information and transfer entropy*.

We predicted the region where an order-disorder phase transition began according to a set of criteria of information metrics and compared the predicted region with wavebreak locations (Fig. 5). We located 132 wavebreaks (red dots) that initiated the first spiral wave in the system out of 56 combinations of train of stimuli (SI Appendix 6). We used only the wavebreaks that initiated the first spiral wave because the first spiral wave induced a chain reaction of breaking up into multiple spiral waves^46^. This reaction is an intrinsic cardiac dynamics that is independent of the interaction between the driving component and each component of the lattice (SI Movie 7). The spatial distribution of the wavebreak locations (red dots) clearly demonstrates a high repeatability of 56 independently executed simulations.

Because channel capacity reached zero at the order-disorder phase transition (Fig. 4C), we used it as a criterion of prediction. Channel capacity of wavebreak locations immediately prior to the phase transition was a non-zero value. Because the non-zero value was unknown *a priori*, we used the maximum channel capacity (=2.8 × 10^−3^ nats) out of all the wavebreak locations immediately prior to the phase transition (BCL = 219 msec) as a cutoff of channel capacity under which a wavebreak could occur. Figure 5A shows a predicted region of phase transition (white) and wavebreak locations (red) in an error space and in a physical space. The error space showed that the predicted region (white) had higher channel capacity than most of the wavebreak locations (Fig. 5A, left two columns). The physical space shows that channel capacity sharply demarcates a circular border across which the wavebreaks occurred (Fig. 5A, right column). Of note, the predicted region (white) was located upstream of most of the wavebreaks (red) with respect to the stimulus site. This indicates that channel capacity can define a geometrical border which serves as a frontline of action. Since wavebreaks occurred as soon as the wave traveled across the border, having the predicted region upstream of wavebreaks has an important therapeutic implication for mitigating the onset of phase transition by modifying the components at the border.

Likewise, we used mutual information as another criterion to predict the region of the phase transition. We used the maximum mutual information (=2.5 × 10^−3^ nats) out of all the wavebreak locations immediately prior to the phase transition (BCL = 219 msec) as a cutoff of mutual information under which a wavebreak can occur. As expected from the 2-D dynamics of mutual information, the result was almost identical to that of channel capacity in both the error space and the physical space (Fig. 5B).

We also used transfer entropy as an additional criterion of prediction. Since transfer entropy is known to peak prior to a phase transition^31^, we used the minimum transfer entropy (=7.3 × 10^−4^ nats) out of all the wavebreak locations immediately prior to the phase transition (BCL = 219 msec) as a cutoff of transfer entropy under which a wavebreak could occur. Figure 5C shows a predicted region of phase transition (white) and wavebreak locations (red) in an error space and in a physical space. The error space shows that the predicted region (white) has lower channel capacity than some of the wavebreak locations (Fig. 5C, left two columns). The physical space shows that transfer entropy also demarcates a circular border across which the wavebreaks occur, similar to that of the channel capacity criterion (Fig. 5C, right column). Importantly, the predicted region (white) is immersed in the middle of most of the wavebreaks (red), slightly downstream of the border by the channel capacity criterion. This indicates that, in addition to channel capacity, transfer entropy can separately define a geometrical border across which phase transitions can occur.

To highlight the difference among those three criteria, Fig. 5D shows the predicted region using either the channel capacity, mutual information, or the transfer entropy criterion. Both the error space (Fig. 5D, left two columns) and the physical space (Fig. 5D, right column) clearly shows that the regions of phase transition predicted by those information metrics match well with the wavebreak locations. However, there is a slight difference between channel capacity/mutual information and transfer entropy in both the error space and the physical space. Importantly, in the physical space, those criteria define different circular borders. This finding indicates that, although both channel capacity/mutual information and transfer entropy can be used to predict phase transitions, they represent different underlying information dynamics leading to the transition.

## Discussion

Using information theory as a guiding principle to describe cardiac dynamics, we successfully simulated an order-disorder phase transition in a cardiac system with an interval between regular stimuli as a control parameter (Fig. 2). Our main findings are summarized as follows:

1. Channel capacity, mutual information, and transfer entropy can predict geometrical borders beyond which an order-disorder phase transition from a regular heart rhythm to fibrillation occurs in a cardiac system.
2. Channel capacity and mutual information progressively decline and reach zero at the border of phase transition. This indicates that those information-theoretic metrics can serve as order parameters to describe the macroscopic behavior of the system. Importantly, mutual information can be used as an order parameter in systems where computation of channel capacity is not straightforward because the channel cannot be modeled as a binary asymmetric channel.
3. Mutual information is lower than, but is consistently close to channel capacity, which indicates that the channel is operating close to the maximum capacity, and the cardiac coding sequences are nearly optimized when the cardiac communication channel is modeled as a binary asymmetric channel.
4. Transfer entropy peaks prior to the phase transition at a wide range of BCL. This finding suggests that there is a complex and dynamic information transaction between the stimulus site and individual cardiac components.
5. A period-doubling bifurcation precedes the phase transition to fibrillation, and the BCL at bifurcation is spatially heterogeneous due to the node behavior (Fig. 3). This finding confirms that even a simple, anatomically homogeneous model can be functionally heterogeneous in a cardiac system due to intrinsic dynamic properties such as restitution, wavefront curvature and conduction velocity. This finding also demonstrates that functional heterogeneity alone is a sufficient substrate for regional heterogeneity of period-doubling bifurcation, as opposed to anatomical^47^ or cellular heterogeneity^48^. In addition, the spatial heterogeneity of APD alternans may account for its lack of sensitivity and specificity as a marker of sudden cardiac death^41^ due to the limitation of accurately quantifying APD alternans using clinically available systems.
6. A period-doubling bifurcation, APD alternans, or even a refractory (2:1) response does not significantly impact the cardiac macrostate. Shannon entropy shows a small and steady decline across the bifurcation as BCL goes down (Figs 3 and 4). This finding suggests that – although a period-doubling bifurcation in cardiac systems denotes a qualitative change in the behavior of the system as a function of BCL – the bifurcation does not change macroscopic behaviors. This further suggests that a period-doubling bifurcation and APD alternans alone do not contribute to an order-disorder phase transition from a regular heart rhythm to fibrillation in a cardiac system.

Studies report that mutual information between all-to-all pairs of components in the system peaks precisely at the phase transition in many complex systems, including the Ising model^25,26^, a swarm model^27,28^, random Boolean networks^29^, and financial markets^30^. When the system is highly ordered, little uncertainty about the state of individual components makes mutual information small. In contrast, when the system is highly disordered, mutual information is also small because the components behave almost independently. The mutual information is maximum at the phase transition where susceptibility peaks^31^. In contrast, in the present study, mutual information and channel capacity did not peak at the order-disorder phase transition. Instead, they changed from non-zero to zero at the transition from the ordered side to the disordered side. This is because those metrics in our approach were essentially evaluating how well two components were communicating with each other. This novel approach is characterized by three advantages. First, our approach allows channel capacity and mutual information as order parameters to quantify the macroscopic behaviors of the system. The phase transition occurs at the weakest link of communication, or miscommunication. Second, those information-theoretic metrics help predict order-disorder phase transitions, because they become zero at the transition. This highlights the advantage of our approach over traditional approaches where mutual information does not help predict phase transitions because it peaks precisely at transition. Third, our approach is computationally efficient even in a system with large numbers of components.

The role of transfer entropy in predicting order-disorder phase transitions is less characterized than that of mutual information. In random Boolean networks, a phase transition is characterized by the shifting balance of local information storage over transfer^49^. In the Ising model, a collective multivariate transfer entropy – global transfer entropy – peaks on the disordered side of a transition^31^. Global transfer entropy indicates a balance between integration and segregation of complex networks of dynamical processes^50–52^. The mechanism of these results remains unclear, but it is relevant to many natural and social systems where disorder is associated with healthy features and order with pathological dynamics, such as synchronization in epileptic seizures and herding behaviors in financial market crashes^53^. In contrast, in the heart, order is associated with healthy dynamics and disorder is associated with pathological, often lethal, dynamics. In our data, we found that the transfer entropy peaked on the ordered side of a transition. In particular, the transfer entropy, which peaked beyond the period-doubling bifurcation, was found to help identify the locations of phase transition initiation. It is possible that transfer entropy peaks between the period-doubling bifurcation and the phase transition at wavebreaks uncovers complex interactions between the driving component and the local components at wavebreaks near criticality.

Implantable cardioverter-defibrillators (ICD) are the standard of care for primary prevention of sudden cardiac death in high-risk patients who are yet to experience fatal events^54^. However, only a minority of ICD recipients experience appropriate firings based on the current criteria for primary prevention ICD, relying on impaired cardiac function assessed by functional class and left ventricular ejection fraction^55^. In addition, the cost and the risks of complications^56^ and inappropriate firings^57^ do not warrant indiscriminate application of ICD therapy. Our approach provides an alternative strategy by identifying and targeting the cardiac components with interventional catheter ablation therapies to prevent sudden death resulting from ventricular fibrillation^18–20^. Our approach is potentially applicable to other clinical conditions such as epileptic seizures^58^ in which spiral waves play a major role. Application of this approach could also have relevance to a wide range of systems to mitigate or prevent the imminent order-disorder phase transition by identifying and eliminating the component responsible for the initiation of the transition.

We recognise several limitations associated with the numerical method we implemented. We used a relatively simple cardiac model^59^, with a homogeneous and isotropic 2-D lattice. It is possible that a more biophysically detailed model of the heart with anatomical heterogeneity, anisotropy and a more realistic geometry could make our approach more difficult to analyse. For example, the functional heterogeneity that we observed may further be accentuated in a more realistic model, making the analysis more vulnerable to noise. However, the simplicity of the cardiac model is an advantage that allows the results from this model to be widely applicable to other complex systems to gain general insights as to how information-theoretic metrics help locate phase transitions. In addition, the focus of the present work is information-theoretic analysis of dynamic properties of the cardiac system that leads to an order-disorder phase transition in structurally normal hearts, such as paroxysmal atrial fibrillation and idiopathic ventricular fibrillation. Therefore, the applicability of our approach to cardiac systems with structural abnormality involving cardiomyopathy and myocardial fibrosis remains to be seen. Furthermore, we did not simulate the Purkinje fibers with faster conduction within the lattice. Idiopathic ventricular fibrillation is usually triggered by a train of ectopic beats from the Purkinje fibers, but the mechanism as to how those Purkinje-derived ectopies generate wavebreaks remains unclear^16^. Our relatively simple model of cardiac system clearly demonstrates that a train of ectopic beats can cause wavebreaks and fibrillation in the absence of the Purkinje fibers within the lattice. This suggests that faster conduction in the Purkinje fibers is not a necessary condition, but dynamic properties of the ordinary myocardium alone are sufficient for fibrillation to occur. This finding is consistent with the clinical observation that successful interventional catheter ablation of idiopathic ventricular fibrillation does not require extensive Purkinje fiber perturbation, but only focal targeting of the origin of the train of ectopic beats in the Purkinje fiber. This finding is also consistent with the pathogenesis of paroxysmal atrial fibrillation in the atria where the Purkinje fibers with faster conduction are absent. In fact, slower conduction is more favorable for sustaining functional reentry of fibrillation, because the “*effective size*” of the myocardium is larger with slower conduction^60^.

In conclusion, we developed an information-theoretic approach to predict the locations of an order-disorder phase transition from a regular heart rhythm to fibrillation in a cardiac system. Our computationally efficient approach is applicable not only to the cardiac system but also to a wide range of systems in distinct physical, chemical and biological systems.

## Methods

We performed the simulation and the data analysis using Matlab R2017a (Mathworks, Inc.).

### Model of cardiac system

We used a deterministic, simplified ionic model of the cardiac action potential described by Fenton and Karma^59^. We chose this model because it accurately reproduces the critical properties of the cardiac action potential to test our hypothesis, such as restitution properties, APD alternans, conduction block, and spiral wave initiation^46^. The model consists of three variables: the transmembrane potential *V*, a fast ionic gate *u*, and a slow ionic gate *w*.

1 $$\frac{\partial V}{\partial t}=\nabla \cdot (D\nabla V)-\frac{{I}_{fi}+{I}_{so}+{I}_{si}+{I}_{ex}}{{C}_{m}}$$

Here *C*_*m*_ is the membrane capacitance (=1 *μF*/*cm*^2^), and *D* is the diffusion tensor, which is a diagonal matrix whose diagonal and off-diagonal elements are 0.001 cm^2^/msec and 0 cm^2^/msec, respectively, to represent a 2-D isotropic system^46^. The current *I*_*fi*_ is a fast inward inactivation current used to depolarize the membrane when an excitation above threshold is induced. The current *I*_*so*_ is a slow, time-independent rectifying outward current used to repolarize the membrane back to the resting potential. The current *I*_*si*_ is a slow inward inactivation current used to balance *I*_*so*_ and to produce the observed plateau in the action potential. *I*_*ex*_ is the external current^61^ (SI Appendix 1).

We solved the model equations with an explicit Euler integration at Δ*t* = 0.1 msec. For 1-D and 2-D simulations, we used a finite difference method for spatial derivatives (Δ*x* = 0.025 cm) assuming Neumann boundary conditions. This set of parameters satisfies von Neumann stability requirement for 1-D and 2-D finite difference schemes.

2 $$\frac{D{\rm{\Delta }}t}{{({\rm{\Delta }}x)}^{2}}\le \frac{1}{2d}$$

where *d* = 1 for 1-D and *d* = 2 for 2-D simulations.

The interval between regular stimuli with an external current, or basic cycle length (BCL), ranged between 200 msec and 300 msec. We applied 220 stimuli for 1-D and 60 stimuli for 2-D simulations at each BCL to reach a steady state prior to progressively reducing the BCL by a decrement of 1 msec to achieve the minimum effective refractory period.

### Cardiac simulation

For 1-D simulations we stimulated the origin (*x* = 0) of the system of a 1-D cable of length 40 cm and acquired the time series of 60 beats in each component at each BCL. For 2-D simulations, we stimulated the top left component of the system of a 2-D lattice of 25 cm × 25 cm and acquired the time series of 60 beats in each component at each BCL. This size is relatively large for a human heart, but we found that this level of surface area is required for this specific model of cardiac system to induce a sufficient number of order-disorder phase transitions to validate our information-theoretic approach^62,63^. The time series were downsampled to achieve the final sampling frequency of 1 kHz (Δ*t* = 1 msec) to reflect realistic measurements in human clinical electrophysiology studies^64^.

### Restitution properties

We calculated APD, CV and diastolic interval (DI) at each BCL several points away from the stimulus site in the 1-D cable to avoid stimulus artifacts and boundary effects^46^. APD was measured at 90% repolarization (*APD*_90_) at a steady state at each component. The *i*th diastolic interval (*DI*_*i*_) is defined as the difference between BCL and the *i*th *APD* (*APD*_*i*_) (Fig. 1A):

3 $$D{I}_{i}=BCL-AP{D}_{i}$$

We used local regression with weighted linear least squares and a first-degree polynomial model to determine CV at each BCL, because it depends sensitively on the time measurements^46^.

### APD alternans, curvature, and wavebreak

For both 1-D and 2-D simulations, the period-doubling bifurcation was defined as the longest BCL that generates APD alternans at a steady state. APD alternans was defined to be present when APD alternates between the longer and the shorter APD in response to regular stimuli, and the difference between the longer and the shorter APD is greater than 5 msec. For 2-D simulations, curvature of the wavefront was defined as the reciprocal of the radius of its osculating circle at each component and was described in arbitrary units. When the stimuli generate wavebreaks that initiate spiral waves, we identified the coordinates of wavebreaks in the 2-D lattice. The wavebreak was defined as the point of maximum curvature intersecting the wavefront and the waveback that initiates the first spiral wave in each time series. There are typically 2–6 wavebreaks per time series.

### Information-theoretic metrics

The time series of cardiac excitation in each component was encoded as 1 when excited (during *APD*_90_) and 0 when resting^33^ (Fig. 1A). The conduction delay to travel from the stimulus site to each component was subtracted from the time series to allow comparison of the same traveling wave at each component. We treated each component as a time-series process *X*, where at any observation time *t* the process *X* is either excited (*X* = 1) or resting (*X* = 0). We considered the stimulus site as an information sender/encoder with an input *X*, and any other component in the system as a receiver/decoder with an output *Y* (Fig. 1B). We considered the intervening components between the sender/encoder and the receiver/decoder as channels.

The Shannon entropy *H* of each time-series process *X* is given by

4 $$\begin{array}{rcl}H(X) & = & -\sum _{x}\,p(x)\,{\mathrm{log}}_{2}\,p(x)\end{array}$$

5 $$\begin{array}{rcl} & = & -p(X=\mathrm{0)}\,{\mathrm{log}}_{2}\,p(X=\mathrm{0)}-p(X=\mathrm{1)}\,{\mathrm{log}}_{2}\,p(X=\mathrm{1)}\end{array}$$

where *p*(*x*) denotes the probability density function of the time series generated by *X*. This quantifies the average uncertainty of whether a single component is excited (*x* = 1) or resting (*x* = 0) over the time history^65^.

The channel capacity *C* between the input *X* and the output *Y* is given by

6 $$C=\mathop{{\rm{\max }}}\limits_{p(x)}\,I(X;Y)$$

where *I*(*X*;*Y*) is mutual information that quantifies the information content shared between the input *X* and the output *Y*.

7 $$I(X;Y)=\sum _{x,y}\,p(x,y)\,{\mathrm{log}}_{2}\frac{p(x,y)}{p(x)p(y)}$$

where *p*(*x*, *y*) denotes the joint probability density function of *X* and *Y*. We considered these channels to be a binary asymmetric channel, which is the most general form of binary discrete memoryless channel (Fig. 1C). The channel has a probability *ε*_0_ that an input 0 will be flipped into a 1 (type 0 error) and a probability *ε*_1_ for a flip from 1 to 0 (type 1 error).

8 $$p(Y=0|X=\mathrm{0)}=1-{\varepsilon }_{0}$$

9 $$p(Y=1|X=\mathrm{0)}={\varepsilon }_{0}$$

10 $$p(Y=1|X=1)=1-{\varepsilon }_{1}$$

11 $$p(Y=0|X=1)={\varepsilon }_{1}$$

The channel capacity of the binary asymmetric channel is (SI Appendix 2)

12 $$C={\mathrm{log}}_{2}\,(1+z)-\frac{1-{\varepsilon }_{1}}{1-{\varepsilon }_{0}-{\varepsilon }_{1}}h({\varepsilon }_{0})+\frac{{\varepsilon }_{0}}{1-{\varepsilon }_{0}-{\varepsilon }_{1}}h({\varepsilon }_{1})$$

where

13 $$z={2}^{\frac{h({\varepsilon }_{0})-h({\varepsilon }_{1})}{1-{\varepsilon }_{0}-{\varepsilon }_{1}}}$$

The transfer entropy^24^ is a non-parametric statistic measuring the directed reduction in uncertainty in one time-series given another, generally interpreted as information transfer. The transfer entropy from the input *X* to the output *Y* is the amount of uncertainty reduced in future values of *Y* by knowing the past values of *X*, given past values of *Y*^66^.

14 $$\begin{array}{rcl}{T}_{X\to Y} & = & \sum \,p({y}_{t+1},{y}_{t}^{l},{x}_{t}^{k})\,{\mathrm{log}}_{2}\frac{p({y}_{t+1}|{y}_{t}^{l},{x}_{t}^{k})}{p({y}_{t+1}|{y}_{t}^{l})}\end{array}$$

15 $$\begin{array}{rcl} & = & H({y}_{t+1}|{y}_{t}^{l})-H({y}_{t+1}|{y}_{t}^{l},{x}_{t}^{k}),\end{array}$$

where *k* and *l* denote the length of time series in the processes *X* and *Y*, respectively:

16 $${x}_{t}^{k}=({x}_{t},{x}_{t-1},\ldots ,{x}_{t-k+1})$$

17 $${y}_{t}^{l}=({y}_{t},{y}_{t-1},\ldots ,{y}_{t-l+1}).$$

We define *k* and *l* such that ${x}_{t}^{k}$ and ${y}_{t}^{l}$ contain the entire time-series (*k* = *l*). We used the discrete transfer entropy calculator of the Java Information Dynamics Toolkit (JIDT) to calculate transfer entropy^67^.

We adopt the standard convention of 0 · log_2_ 0 = 0.

### Data availability

All data generated and/or analysed during the present study are available in the following link: https://jh.box.com/s/0w4u455qesz948lkt8mnpo4wpxesia78.

## Electronic supplementary material

SI Movie 5

SI Movie 6

SI Movie 7

SI Movie 1

SI Movie 2

SI Movie 3

SI Movie 4

Supplementary Information
