# Supplementary material for: Locating Order-Disorder Phase Transition in a Cardiac System
Source: Sci Rep. 2018 Jan 31;8:1967. doi: 10.1038/s41598-018-20109-6 (PMC5792589; doi:10.1038/s41598-018-20109-6)
Supplement: Supplementary file 8 — Supplementary Information [file 41598_2018_20109_MOESM8_ESM.pdf]

Supplementary Information for

Locating Order-Disorder Phase Transition in a Cardiac System

Hiroshi Ashikaga* and Ameneh Asgari-Targhi

*To whom correspondence should be addressed. E-mail: hashika1@jhmi.edu

SI Appendix 1. Restitution properties

The APD restitution curve defines how the length of consecutive 0s (= diastolic interval, DI) determines the length of the next consecutive 1s (= APD) in the binary sequence of cardiac microstate. At BCL=300 msec, APD converged to a stable fixed point (APD=185 msec) after several beats (Figure 1A). At BCL=240 msec, the APD response showed classic oscillatory dynamics of APD alternans with long (= 184 msec) and short APD (= 128 msec)(Figure 1B). At BCL=200 msec, every other stimulus could not evoke APD because the component was refractory, resulting in a 2:1 response (Figure 1C). Overall, the model successfully reproduced the nonlinear bifurcation behavior of cardiac systems. As the BCL progressively declined, the slope APD/DI increased and approached 1 (Figure 1D, dashed line), when the cardiac microstate suddenly changed due to a period-doubling bifurcation. This corresponds to BCL=248 msec, beyond which APD alternans was observed (Figure 1E, dashed line). Shannon entropy showed a bifurcation when it is plotted against DI (Figure 1F, dashed line). This simply shows that one value of DI larger than the bifurcation represents two different cardiac macrostates that derive from two different BCLs due to APD alternans. When Shannon entropy is plotted against BCL, it showed a small and steady decline across the bifurcation as BCL decreased (Figure 1G, dashed line). This indicates that the period-doubling bifurcation does not impact the cardiac macrostate – a probability distribution over the possible microstates. The model also successfully reproduced the conduction velocity (CV) restitution curve, which defines how the length of consecutive 0s (= DI) determines the delay of communication between two components (Figure 1D). CV was relatively flat for a wide range of DI, and only began to decrease at a very short DI (approximately 100 msec).

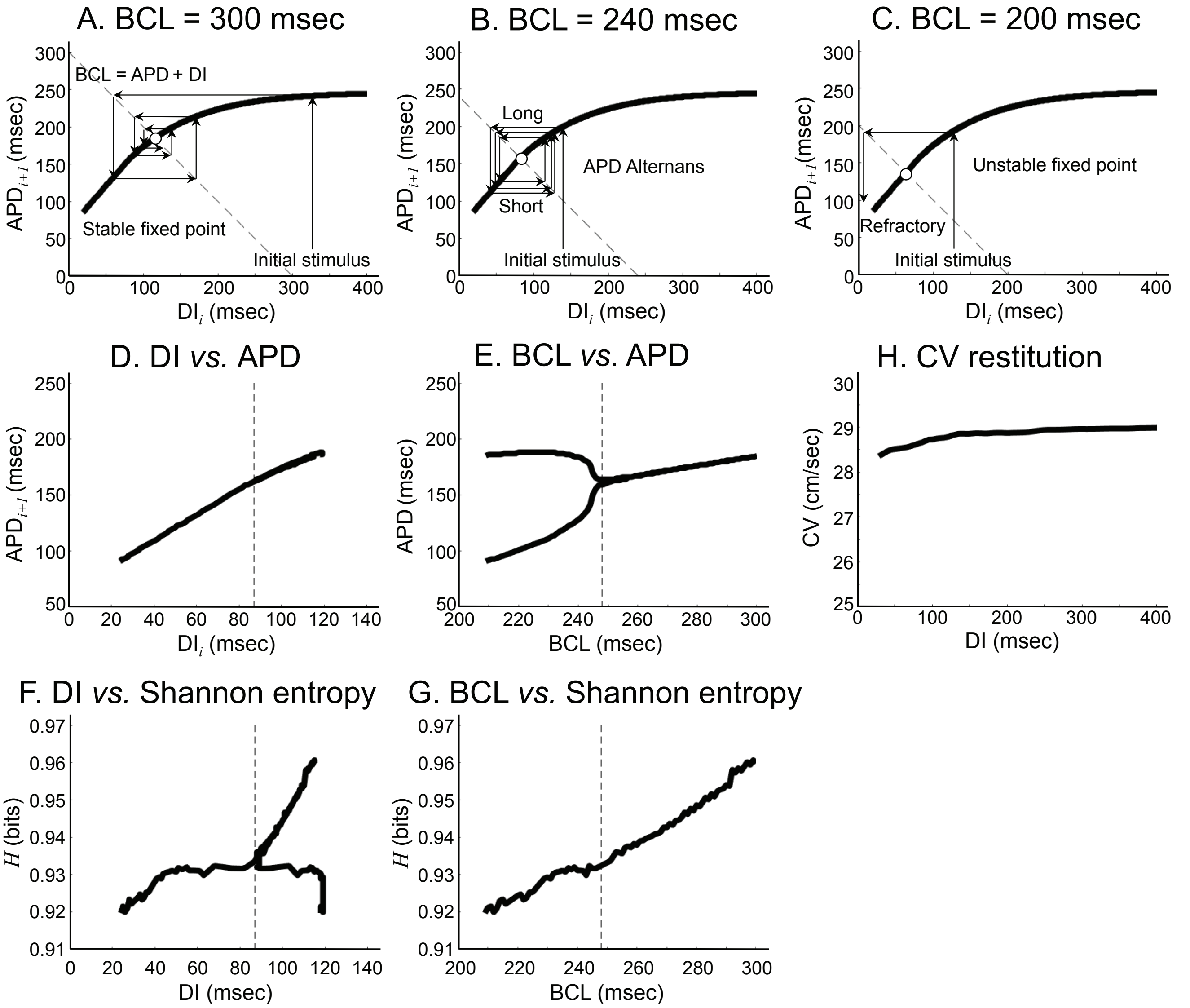

Figure 1. Restitution properties. *A. BCL=300 msec (normal response).* The solid curve shows the APD restitution curve; the dashed line shows the relationship between APD and DI for the new BCL (BCL=APD+DI). Over several stimuli, APD converged to a stable fixed point (APD=185 msec). *B. BCL=240 msec (APD alternans).* The APD response shows oscillatory dynamics alternating with long (APD=184 msec) and short APD (APD=128 msec). *C. BCL=200 msec (refractory response).* The APD response to the initial stimulus led to DI shorter than the minimal value in the APD restitution curve. This resulted in a 2:1 response. Figure modified from [1]. *D, E, F, and G.* A zoomed view of the steeper end of the APD restitution curve during BCL 200-300 msec. The dashed line shows the period-doubling bifurcation (BCL=248 msec). *F* and *G* show Shannon entropy as a function of DI and BCL, respectively. *H. Conduction velocity (CV) restitution curve.*

SI Appendix 2. APD alternans and conduction block in 1-D cable

At BCL=300 msec, one stimulus generated one action potential, and APD was uniform spatially and temporally (APD=185 msec, Figure 2A, *SI Movie 1*). Shannon entropy was consistently high throughout the cable length (Figure 2E), which indicates that the cardiac macrostate is homogeneous across the cable. Channel capacity and mutual information were also consistently high. Mutual information was the lowest of the three information metrics but was close to channel capacity, which indicates that the channel is operating close to the maximum capacity. Transfer entropy was small and variable with fluctuations across the cable length (Figure 2F).

At BCL=220 msec, one stimulus generated one action potential, but APD at the stimulus site was alternating with long (APD=188 msec) and short APDs (APD=101 msec) (Figure 2B, top row, *SI Movie 2*). At a short distance away from the stimulus site on the cable (Figure 2B, second row), the APD alternans improved and APD became uniform temporally. This is called a node behavior since this site serves as a node to link the regions of *concordant* and *discordant alternans*[2]. At midway of the cable (Figure 2B, third row), APD alternans reappeared but π out of phase from the stimulus site (*discordant alternans*)[3]. At a further distance away from the stimulus site (Figure 2B, fourth row), the APD alternans improved and APD became uniform temporally again, showing another node behavior. At furthest distance away from the stimulus site (Figure 2B, bottom row), the APD alternans reappeared in-phase with the stimulus site (*concordant alternans*)[3]. Shannon entropy remained high and constant throughout the cable length (Figure 2G). Channel capacity was high near the stimulus site (cable length=0 cm) but steeply declined to near zero between cable length=10 cm and 25 cm, representing the region of discordant APD alternans. Beyond cable length=30 cm, channel capacity improved at $>$0.8 bits, reflecting concordant APD alternans. Mutual information followed the trend of the channel capacity, with only slightly lower values. Transfer entropy still fluctuated but declined steadily from the stimulus site and reached the minimum value at the beginning of discordant alternans (cable length around 10 cm), indicating little information transfer from the source to this region (Figure 2H).

At BCL=208 msec, one stimulus generated one action potential with APD alternans at the stimulus site, alternating with long (APD=185 msec) and short APDs (APD=90 msec) (Figure 2C, top row, *SI Movie 3*). At a short distance away from the stimulus site on the cable (Figure 2C, second row), the APD alternans became discordant, and eventually conduction block occurred (Figure 2C, third row). At midway and further, only one APD out of two stimuli was conducted (Figure 2C, fourth and bottom rows). There was a tendency for Shannon entropy to decline toward the site of conduction block, where it increased again and remained high throughout the cable length (Figure 2I). Channel capacity was high near the stimulus site (cable length=0 cm) but steeply decreased to near zero at around cable length=10 cm, representing the region of discordant APD alternans and conduction block. Beyond the site of block, the channel capacity improved only slightly but continued to be low ($<$0.16 bits) throughout the cable. Mutual information followed the trend of the channel capacity, with only slightly lower values. Transfer entropy declined steadily but with fluctuations from the stimulus site and reached the minimum value at the beginning of discordant alternans and conduction block (cable length around 10 cm)(Figure 2J). Beyond the site of conduction block, transfer entropy increased slightly and remained constant throughout the remaining cable length, indicating that the information of stimuli from the source continues to be transmitted to this region despite a 2:1 conduction.

At BCL=200 msec, BCL was shorter than APD, and thus hit the refractory period of the previous APD (Figure 2D, *SI Movie 4*). As a result, only every other stimulus generated one action potential at the stimulus site (2:1 response). Shannon entropy remained high throughout the cable length, indicating that the 2:1 response does not impact the cardiac macrostate (Figure 2K). Channel capacity dropped to approximately a half of that of the origin (around 0.5 bits) and maintained the same level throughout the cable, reflecting the 2:1 response. Mutual information followed the trend of the channel capacity, with only slightly lower values. Transfer entropy remained close to zero throughout the cable length (Figure 2L), indicating little information transfer from the source when a 2:1 response is present.

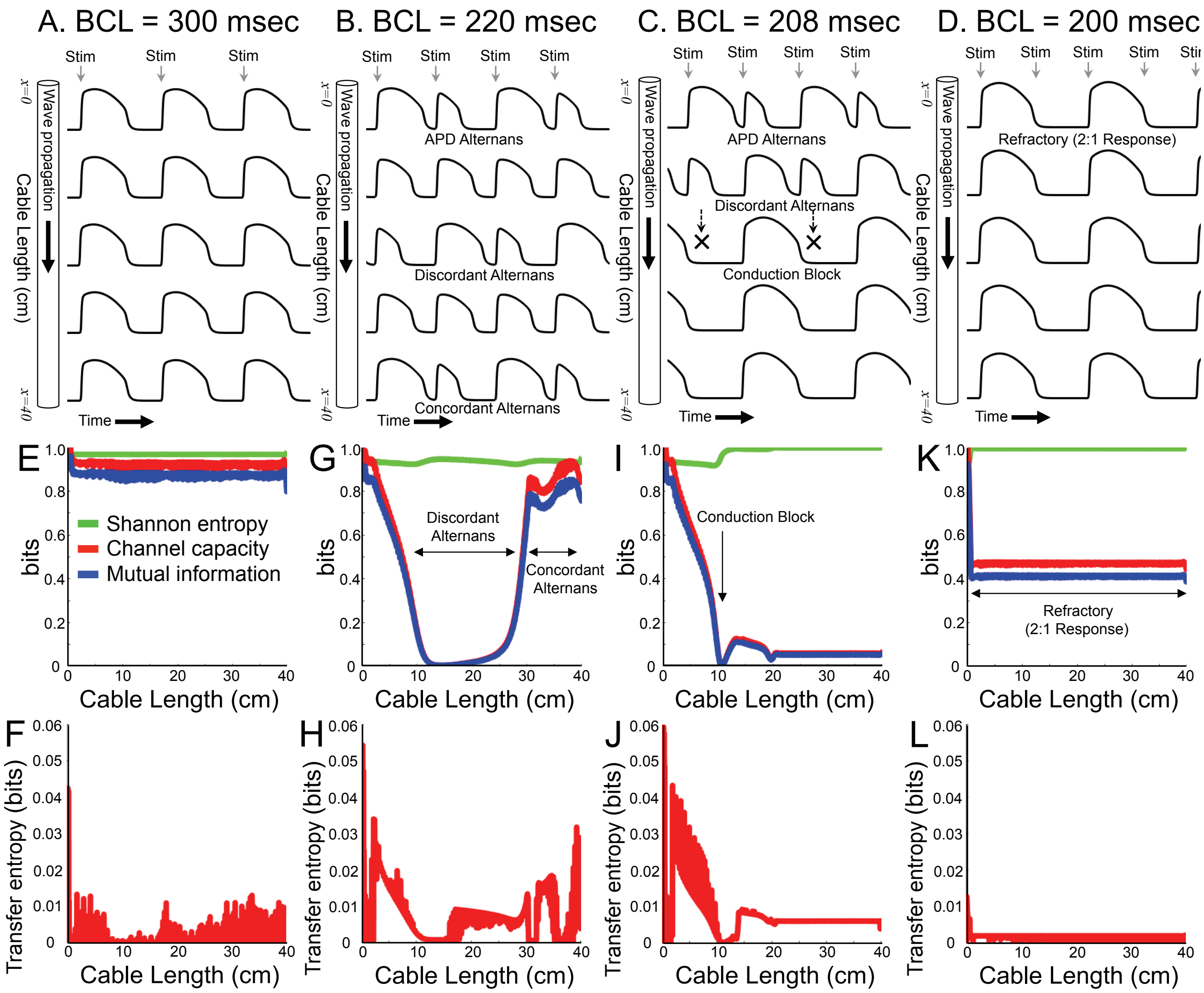

Figure 2. **APD alternans and conduction block in 1-D cable.** *A. BCL=300 msec (normal response).* Waves of cardiac excitation traveled from the stimulus site on the top (x=0 cm) to the bottom (x=40 cm) of a 40-cm, one-dimensional (1-D) cable illustrated on the left hand (Δx = 0.025 cm). *B. BCL=220 msec (APD alternans). C. BCL=208 msec (conduction block). D. BCL=200 msec (refractory response).* Shannon entropy (bits, green line), channel capacity (bits, red line) and mutual information (bits, blue line) are shown as a function of cable length (cm) in *E* (BCL=300 msec), *G* (BCL=220 msec), *I* (BCL=208 msec), and *K* (BCL=200 msec). The stimulus site is at cable length = 0 cm. Transfer entropy (bits, red line) is shown as a function of cable length (cm) in *F* (BCL=300 msec), *H* (BCL=220 msec), *J* (BCL=208 msec), and *L* (BCL=200 msec). The stimulus site is at cable length = 0 cm.

SI Appendix 3. Information dynamics in 1-D cable

Figure 3A-D shows the dynamics of Shannon entropy, channel capacity, mutual information, and transfer entropy as a function of BCL (*x*-axis) and cable length (*y*-axis). Shannon entropy was robust against changes in BCL and the location (Figure 3A). This indicates that BCL has little impact on the cardiac macrostate. We also found that a period-doubling bifurcation was variable depending on the location of the cable (black dashed line). There were three local minima of bifurcation at *x*=7.1 cm (BCL=236 msec), *x*=21.5 cm (BCL=236 msec), and *x*=35.3 cm (BCL=237 msec). Channel capacity showed that those minima of bifurcation were located at the node between concordant and discordant APD alternans, indicating that the variability of bifurcation derives from the node behavior (Figure 3B). To better understand the dynamics of cardiac macrostate as a function of BCL, we conducted an error-space analysis of each component where the *x*-axis is the probability ε_0 that an input 0 will be flipped into a 1 (type 0 error), and the *y*-axis is the probability ε_1 for a flip from 1 to 0 (type 1 error). As BCL declined from 300 msec to 200 msec, the cardiac component at *x*=5 cm started at the origin of the error space and traveled counterclockwise (Figure 3E). Both ε_0 and ε_1 reached a peak where discordant alternans was maximum before BCL hit the refractory period and showed a refractory response. At *x*=20 cm discordant alternans persisted over many BCLs before a conduction block occurred, and eventually a refractory response ensued (Figure 3F). In contrast, at *x*=35 cm the trajectory was initially limited to the vicinity of the origin because concordant alternans occurred more frequently than discordant alternans (Figure 3G). As BCL progressively declined, a conduction block and a refractory response eventually occurred.

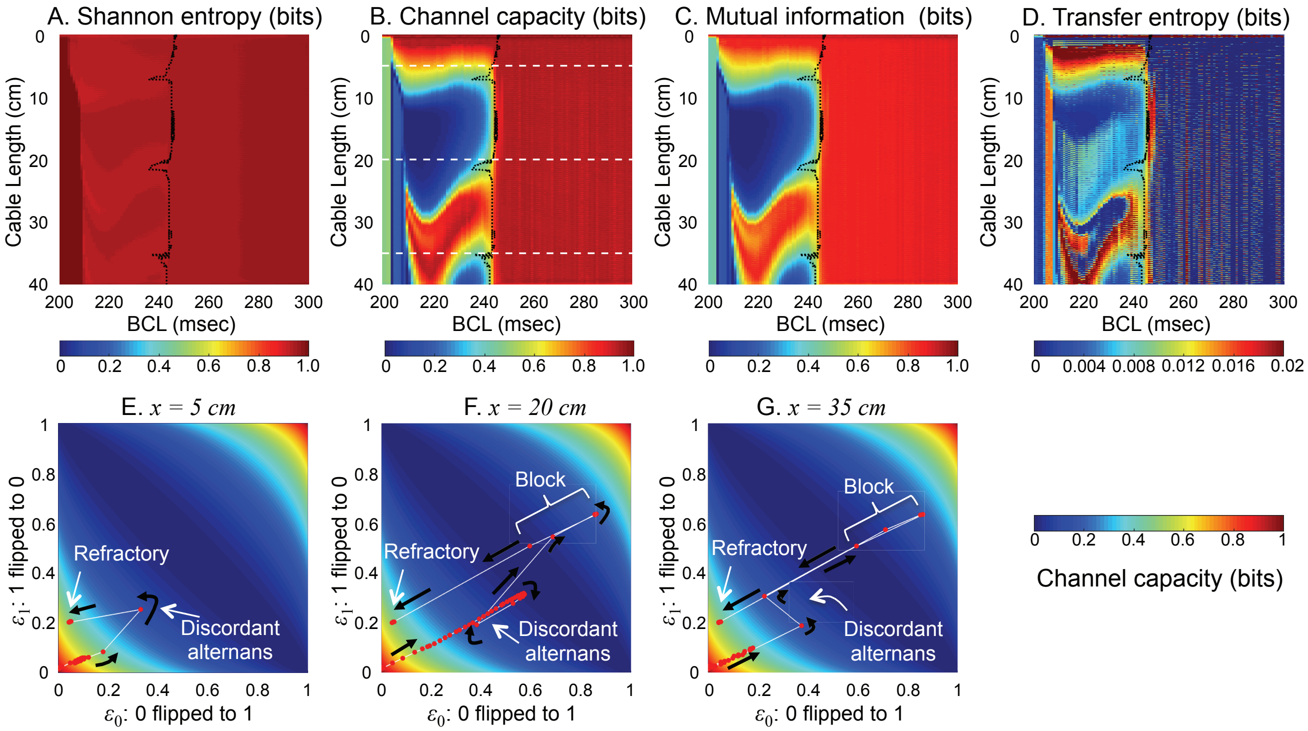

Figure 3. Information dynamics in 1-D cable. *A. Shannon entropy.* Black dashed lines indicate a period-doubling bifurcation at each component with three local minima at *x*=7.1 cm (BCL=236 msec), *x*=21.5 cm (BCL=236 msec), and *x*=35.3 cm (BCL=237 msec). *B. Channel capacity. C. Mutual information. D. Transfer entropy.* White dashed lines in *B* indicate three components shown in the error space analysis, *x*=5cm (*E*), *x*=20cm (*F*), and *x*=35cm (*G*). In the error space, the *x*-axis is the probability ε_0 that an input 0 will be flipped into a 1 (type 0 error), and the *y*-axis is the probability ε_1 for a flip from 1 to 0 (type 1 error). Red dots, white lines and black arrows in the error space indicate the trajectory of each component as BCL goes down from 300 msec to 200 msec.

SI Appendix 4. Fenton-Karma model of cardiac excitation

We used a deterministic, simplified ionic model of the cardiac action potential described by Fenton and Karma[4]. We chose this model because it accurately reproduces the critical properties of the cardiac action potential to test our hypothesis, such as restitution properties, APD alternans, conduction block, and spiral wave initiation[2]. The model consists of three variables: the transmembrane potential V, a fast ionic gate u, and a slow ionic gate w.

$$\frac{\partial V}{\partial t} = \nabla \cdot (D \nabla V) - \frac{I_{fi} + I_{so} + I_{si} + I_{ex}}{C_m} \tag{1}$$

Here C_m is the membrane capacitance (= 1 $\mu F/cm^2$), and D is the diffusion tensor, which is a diagonal matrix whose diagonal and off-diagonal elements are 0.001 cm^2/msec and 0 cm^2/msec, respectively, to represent a 2-D isotropic system[2]. The current I_{fi} is a fast inward inactivation current used to depolarize the membrane when an excitation above threshold is induced. The current I_{so} is a slow, time-independent rectifying outward current used to repolarize the membrane back to the resting potential. The current I_{si} is a slow inward inactivation current used to balance I_{so} and to produce the observed plateau in the action potential. I_{ex} is the external current[5]. The two gate variables of the model follow first order equations in time.

$$\frac{\partial u}{\partial t} = \frac{(1-p)(1-u)}{\tau_u^-} - \frac{pu}{\tau_u^+} \tag{2}$$

$$\frac{\partial w}{\partial t} = \frac{(1-p)(1-w)}{\tau_w^-} - \frac{pw}{\tau_w^+} \tag{3}$$

where

$$\tau_u^- = (1-q)\tau_{u1}^- + q\tau_{u2}^- \tag{4}$$

and

$$p = \begin{cases} 1 & \text{if } V \geq V_c \\ 0 & \text{if } V < V_c \end{cases} \tag{5}$$

$$q = \begin{cases} 1 & \text{if } V \geq V_u \\ 0 & \text{if } V < V_u \end{cases} \tag{6}$$

The transmembrane potential V and the two gate variables u and w vary from 0 to 1. The three currents are given by the following:

$$I_{fi} = -\frac{up}{\tau_d}(V - V_c)(1 - V) \tag{7}$$

$$I_{so} = \frac{V}{\tau_0}(1-p) + \frac{p}{\tau_r} \tag{8}$$

$$I_{si} = -\frac{w}{2\tau_{si}}(1 + \tanh(k(V - V_c^{si}))) \tag{9}$$

We chose the following model parameters to produce action potential dynamics of interest for the study, including APD alternans, conduction block and wavebreak generation: τ_u^+=3.33; τ_{u1}^-=15.6; τ_{u2}^-=5; τ_w^+=350; τ_w^-=80; τ_d=0.407; τ_0=9; τ_r=34; τ_{si}=26.5; k=15; V_c^{si}=0.45; V_c=0.15; and V_u=0.04[2].

SI Appendix 5. Channel capacity for binary asymmetric channels

The channel capacity C between the input X and the output Y is given by

$$C = \max_{p(x)} I(X;Y) \tag{10}$$

where $I(X;Y)$ is mutual information that quantifies the information content shared between the input X and the output Y.

$$I(X;Y) = \sum_{x,y} p(x,y) \log_2 \frac{p(x,y)}{p(x)p(y)} \tag{11}$$

where $p(x,y)$ denotes the joint probability density function of X and Y. We consider these channels to be a binary asymmetric channel, which is the most general form of binary discrete memoryless channel. The channel has a probability ε_0 that an input 0 will be flipped into a 1 (type 0 error) and a probability ε_1 for a flip from 1 to 0 (type 1 error).

$$p(Y=0|X=0) = 1-\varepsilon_0 \tag{12}$$
$$p(Y=1|X=0) = \varepsilon_0 \tag{13}$$
$$p(Y=1|X=1) = 1-\varepsilon_1 \tag{14}$$
$$p(Y=0|X=1) = \varepsilon_1 \tag{15}$$

If $p(X=0) = x$ and $p(X=1) = 1-x$,

$$p(Y=0) = (1-\varepsilon_0)p(X=0) + \varepsilon_1 p(X=1) \tag{16}$$
$$= (1-\varepsilon_0-\varepsilon_1)x + \varepsilon_1 \tag{17}$$

The conditional entropy of Y given X is

$$H(Y|X) = xh(\varepsilon_0) + (1-x)h(\varepsilon_1) \tag{18}$$

where h is the binary entropy function defined as

$$h(p) = -p\log_2 p - (1-p)\log_2(1-p) \tag{19}$$

The mutual information as a function of x is

$$I(x) = I(X;Y) = H(Y) - H(Y|X) \tag{20}$$
$$= h[(1-\varepsilon_0-\varepsilon_1)x+\varepsilon_1] - xh(\varepsilon_0) - (1-x)h(\varepsilon_1) \tag{21}$$
$$= h[(1-\varepsilon_0-\varepsilon_1)x+\varepsilon_1] - x[h(\varepsilon_0)-h(\varepsilon_1)] - h(\varepsilon_1) \tag{22}$$

The derivative of $I(x)$ is

$$I'(x) = (1-\varepsilon_0-\varepsilon_1)\log_2\left[\frac{1}{x(1-\varepsilon_0-\varepsilon_1)+\varepsilon_1} - 1\right] - [h(\varepsilon_0)-h(\varepsilon_1)] \tag{23}$$

because

$$h'(x) = \log_2\left(\frac{1}{x} - 1\right) \tag{24}$$

Solving $I'(x) = 0$ gives

$$\frac{1}{x(1-\varepsilon_0-\varepsilon_1)+\varepsilon_1} - 1 = z \tag{25}$$

where

$$z = 2^{\frac{h(\varepsilon_0)-h(\varepsilon_1)}{1-\varepsilon_0-\varepsilon_1}} \tag{26}$$

and thus

$$x = \frac{1}{1-\varepsilon_0-\varepsilon_1}\left(\frac{1}{z+1}-\varepsilon_1\right) \tag{27}$$

$$= \frac{1-\varepsilon_1(1+z)}{(1-\varepsilon_0-\varepsilon_1)(1+z)} \tag{28}$$

Therefore, the channel capacity of the binary asymmetric channel is

$$C = h\left[\frac{1-\varepsilon_1(1+z)}{1+z}+\varepsilon_1\right] - \frac{1-\varepsilon_1(1+z)}{(1-\varepsilon_0-\varepsilon_1)(1+z)}\left[h(\varepsilon_0)-h(\varepsilon_1)\right] - h(\varepsilon_1) \tag{29}$$

$$= h\left(\frac{1}{1+z}\right) - \frac{\log_2 z}{1+z} + \varepsilon_1 \log_2 z - h(\varepsilon_1) \tag{30}$$

because

$$h\left(\frac{1}{1+z}\right) = \log_2(1+z) - \frac{z\log_2 z}{z+1} \tag{31}$$

C can be rewritten as

$$C = \log_2(1+z) - \log_2 z + \varepsilon_1 \log_2 z - h(\varepsilon_1) \tag{32}$$

$$= \log_2(1+z) - \frac{1-\varepsilon_1}{1-\varepsilon_0-\varepsilon_1}\left[h(\varepsilon_0)-h(\varepsilon_1)\right] - h(\varepsilon_1) \tag{33}$$

$$= \log_2(1+z) - \frac{1-\varepsilon_1}{1-\varepsilon_0-\varepsilon_1}h(\varepsilon_0) + \frac{\varepsilon_0}{1-\varepsilon_0-\varepsilon_1}h(\varepsilon_1) \tag{34}$$

The input distribution $p(x)$ that maximizes the mutual information and thus achieves the channel capacity is given by[6]

$$p(X=0) = 1 - p(X=1) = \frac{1-\varepsilon_1(1+z)}{(1-\varepsilon_0-\varepsilon_1)(1+z)} \tag{35}$$

SI Appendix 6. Train of stimuli from the driving component to induce order-disorder phase transitions

We used a constant basic cycle length (BCL; $s1$) at 270 msec followed by two extrastimuli ($s2$ and $s3$) to induce order-disorder phase transitions in the cardiac system. Both $s1$ and $s2$ were given for 60 beats each for the system to reach a steady state. An exhaustive search among all possible combinations of train by a decrement of 1 msec revealed 56 combinations that induced order-disorder phase transitions (Figure 4). Outside those 56 combinations, the train either simply did not induce order-disorder phase transitions or resulted in conduction block and/or refractory response because the extrastimuli were too premature.

s3 (msec)																			
220		Block	Block																
219	Block	Block	order																
218	Block	3	order	order															
217	Block	2	7	order															
216	Block	1	6	order	order														
215	Block	Block	5	10	Block	Block	Block						Block	Block					
214		Block	4	9	13	Block	Block	Block	Block	Block	Block	Block	Block	Block	Block	Block			
213		Block	Block	8	12	16	19	Block	Block	Block	Block	Block	43	48	Block	Block			
212			Block	Block	11	15	18	22	25	29	33	37	Block	Block	Block	Block			
211				Block	Block	14	17	21	24	28	32	36	42	47	Block	Block	Block		
210					Block	Block	Block	20	23	27	31	35	41	46	52	Block	Block	Block	
209						Block	Block	Block	Block	26	30	Block	40	45	51	Block	56	Block	
208								Block	Block	Block	Block	34	39	Block	50	Block	55	Block	
207										Block	Block	Block	38	44	Block	Block	54	Block	
206												Block	Block	Block	49	Block	Block	Block	
205													Block	Block	Block	53	Block		
204															Block	Block	Block		
203																Block			
	218	219	220	221	222	223	224	225	226	227	228	229	230	231	232	233	234	235	s2 (msec)

Figure 4. Train of stimuli from the driving component to induce order-disorder phase transitions. The train of stimuli from the driving component to induce order-disorder phase transitions consisted of a constant basic cycle length (BCL; $s1$) at 270 msec followed by two extrastimuli ($s2$ and $s3$). Both $s1$ and $s2$ were given for 60 beats each for the system to reach a steady state. The green cells represent the combination of train ($s1$, $s2$, and $s3$) that induced order-disorder phase transitions, and this system had 56 combinations. The yellow cells ("*order*") represent the combination of train that did not induce order-disorder phase transitions. The black cells represent the combination of train that resulted in conduction block and/or refractory response because the extrastimuli were too premature.

SI Movies

SI Movie 1. BCL = 300 msec in 1-D cable (normal response).
SI Movie 2. BCL = 220 msec in 1-D cable (APD alternans).
SI Movie 3. BCL = 208 msec in 1-D cable (conduction block).
SI Movie 4. BCL = 200 msec in 1-D cable (refractory response).
SI Movie 5. BCL = 300 msec in 2-D lattice (normal response).
SI Movie 6. BCL = 220 msec in 2-D lattice (APD alternans).
SI Movie 7. BCL = 212 msec in 2-D lattice (wavebreaks).

References

1. Gray, R. A. *Theory of rotors and arrhythmias. In Cardiac Electrophysiology: From Cell to Bedside*, chap. 34, 191–223 (Saunders, 2014), 6 edn.

2. Fenton, F. H., Cherry, E. M., Hastings, H. M. & Evans, S. J. Multiple mechanisms of spiral wave breakup in a model of cardiac electrical activity. *Chaos: An Interdiscip. J. Nonlinear Sci.* **12**, 852–892 (2002).

3. Qu, Z., Garfinkel, A., Chen, P.-S. & Weiss, J. N. Mechanisms of discordant alternans and induction of reentry in simulated cardiac tissue. *Circ.* **102**, 1664–1670 (2000).

4. Fenton, F. & Karma, A. Vortex dynamics in three-dimensional continuous myocardium with fiber rotation: filament instability and fibrillation. *Chaos: An Interdiscip. J. Nonlinear Sci.* **8**, 20–47 (1998).

5. Pertsov, A. M., Davidenko, J. M., Salomonsz, R., Baxter, W. T. & Jalife, J. Spiral waves of excitation underlie reentrant activity in isolated cardiac muscle. *Circ. research* **72**, 631–650 (1993).

6. Moser, S. M. Error probability analysis of binary asymmetric channels. *Dept. El. & Comp. Eng., Nat. Chiao Tung Univ* (2009).
